# Supplementary material for: A simple method for mapping the location of cross-β-forming regions within protein domains of low sequence complexity
Source: Proc Natl Acad Sci U S A. 2025 Apr 23;122(17):e2503382122. doi: 10.1073/pnas.2503382122 (PMC12054801; doi:10.1073/pnas.2503382122)
Supplement: Supplementary file 1 — Appendix 01 (PDF) [file pnas.2503382122.sapp.pdf]

Supplementary Data Figures and Legends:

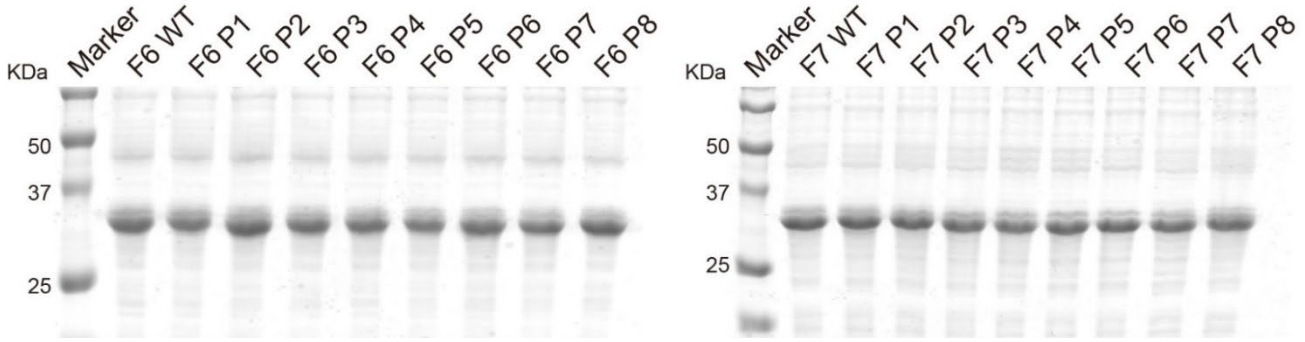

Supplementary Data Figure S1. Coomassie stained SDS-PAGE gels used to visualize fusion proteins linking GFP to either fragment 6 (left panel) or fragment 7 (right panel) of the TDP-43 low complexity domain.

In addition to GFP fusions linked to the native sequences of fragment 6 (F6 WT) and fragment 7 (F7 WT) of the TDP-43 low complexity domain, gel images show GFP fusions linked to double proline variants of each fragment as described in Figure 3 of main text. Molecular weight markers are shown in the left lane of each SDS gel.

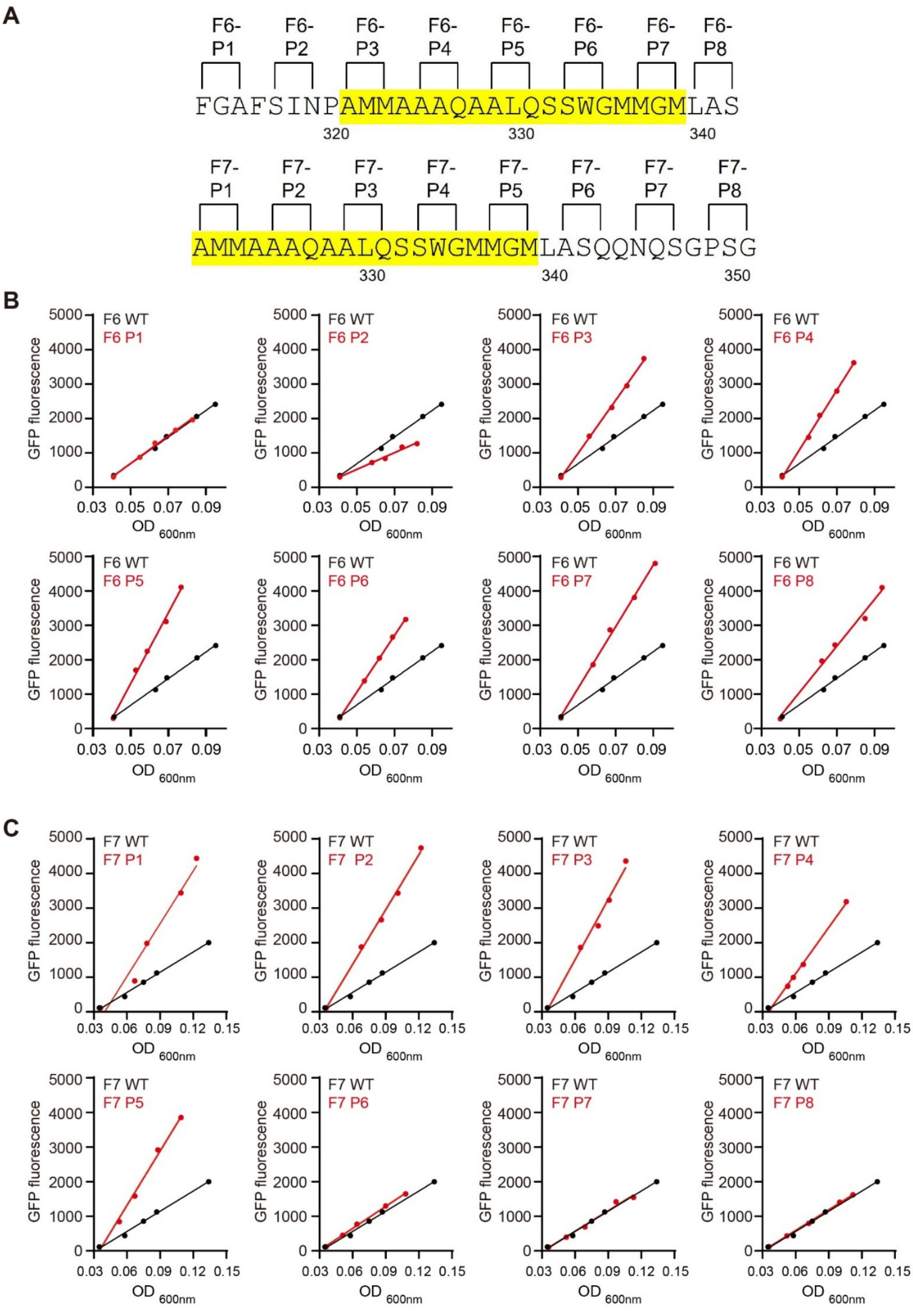
A
F6-P1 F6-P2 F6-P3 F6-P4 F6-P5 F6-P6 F6-P7 F6-P8
FGAFSINPAMMAAAQAALQSSWGMMGMLAS
320 330 340
F7-P1 F7-P2 F7-P3 F7-P4 F7-P5 F7-P6 F7-P7 F7-P8
AMMAAAQAALQSSWGMMGMLASQQNQSGPSG
330 340 350
B
F6 WT
F6 P1
F6 P2
F6 P3
F6 P4
F6 P5
F6 P6
F6 P7
F6 P8
GFP fluorescence
OD 600nm
C
F7 WT
F7 P1
F7 P2
F7 P3
F7 P4
F7 P5
F7 P6
F7 P7
F7 P8
GFP fluorescence
OD 600nm

Supplemental Data Figure S2. Measurements of GFP fluorescence from fusion proteins of the TDP-43 30 residue fragments and double proline variants thereof.

(A) Sequences of fragment 6 (above) and fragment 7 (below) of the TDP-43 low complexity domain with schematic designation of locations of double proline variants. Sequences highlighted in yellow correspond to cross-β forming region. **(B and C)** Measurements of increases in OD_{600nm} as a function of cell density (X axis) plotted against measurements of increases in GFP fluorescence as a function of cell density (Y axis). Black lines for each graph of panel **(B)** describe measurements for cells expressing GFP fused to fragment 6 of the TDP-43 low complexity domain. Black lines for each graph of panel **(C)** describe measurements for cells expressing GFP fused to fragment 7 of the TDP-43 low complexity domain. Red lines of both panels **(B and C)** describe measurements for individual double proline variants. Slope calculations for each double proline variant were used for data shown in Figure 3.

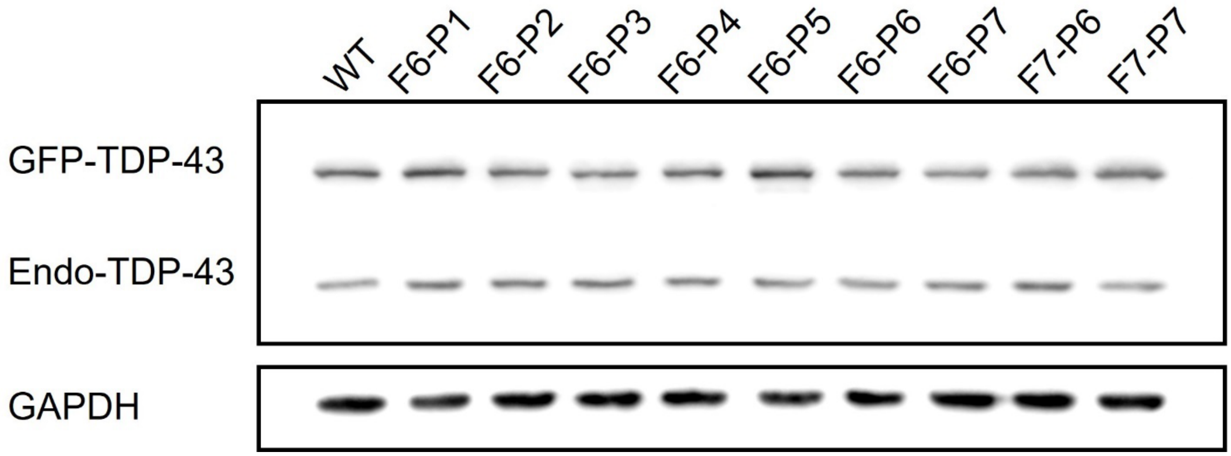

Supplementary Data Figure S3. Western blot assays for the expression of lentivirus-encoded GFP:TDP-43 fusion protein in HCT116 cells.

Top panel shows Western blot of cell lines expressing GFP fusion proteins linked to either the full length, native TDP-43 protein, or to the full length TDP-43 protein bearing designated double proline variants.

The antibody used for top Western blot was specific to the native TDP-43 protein and thus visualized both the exogenous, virus-encoded GFP:TDP-43 fusion protein (GFP-TDP-43) or the endogenous TDP-43 protein of HCT116 cells (Endo-TDP-43). Bottom panel shows Western blot of same samples as assayed in top panel, yet probed with an antibody specific to the GAPDH enzyme endogenous to HCT116 cells.

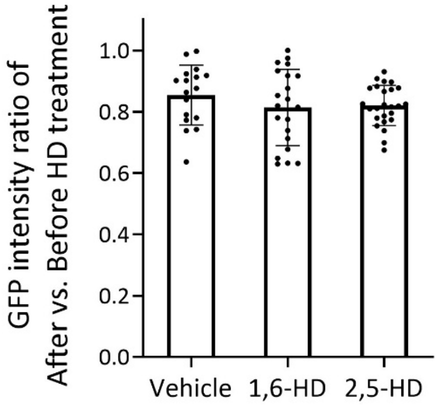

Supplementary Data Figure S4. Exposure of cells to either of two aliphatic alcohols does not significantly alter the background fluorescence of nuclear GFP:TDP-43.

Fluorescence intensity was measured within a 2-µm diameter region devoid of TDP-43 speckles. Treatment was conducted for 5 minutes using either vehicle alone (left), 8 % 1,6-hexanediol (middle), or 8% 2,5-hexanediol (right).

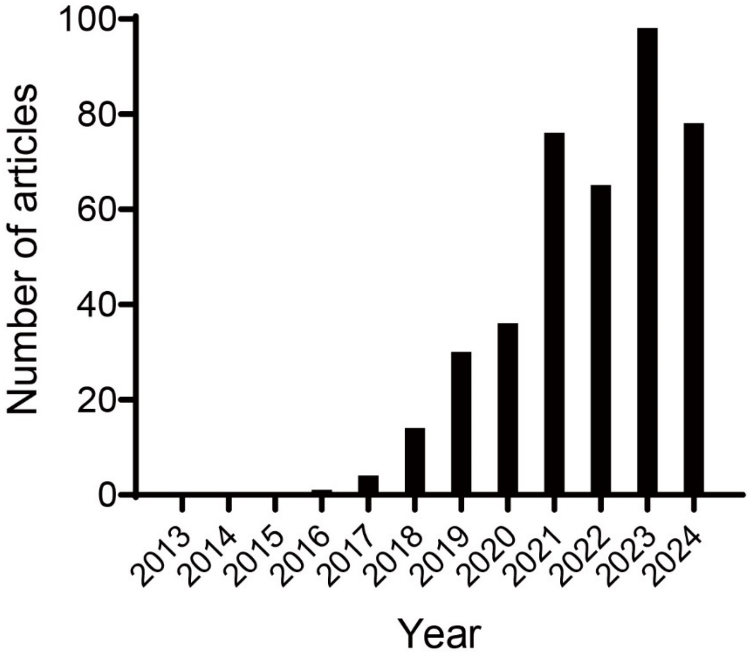

Supplementary Data Figure S5. Google Scholar reported research articles between 2013 and 2024 as identified by all of three key words, *phase separation, 1,6-hexanediol* and *low complexity domain*.
